# Supplementary material for: Vitamin D Modified DSS-Induced Colitis in Mice via STING Signaling Pathway
Source: Biology (Basel). 2025 Jun 18;14(6):715. doi: 10.3390/biology14060715 (PMC12190092; doi:10.3390/biology14060715)
Supplement: Supplementary file 1 [file biology-14-00715-s001.zip › Supplementary Materials S1: 16s-report of gut microbiota/Groups/Lefse_Analysis/out_formant.cladogram.pdf]

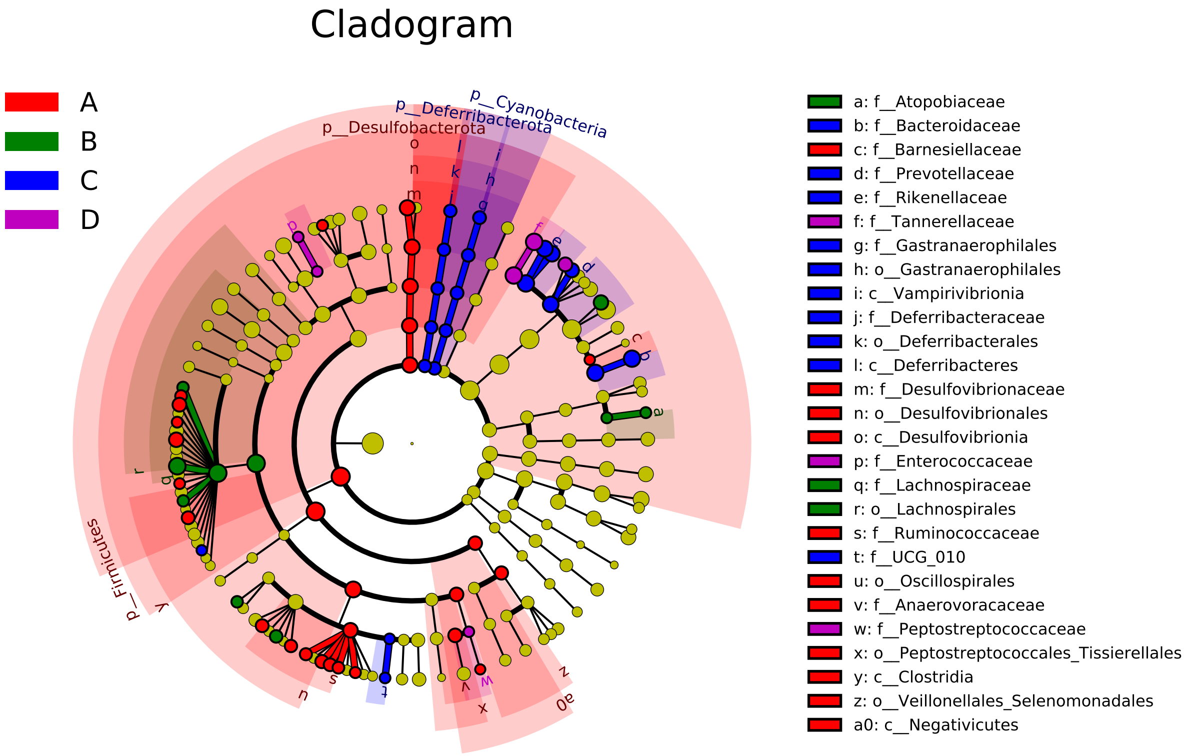

Cladogram
A
B
C
D
p__Desulfobacterota
p__Deferribacterota
p__Cyanobacteria
p__Firmicutes
a: f__Atopobiaceae
b: f__Bacteroidaceae
c: f__Barnesiellaceae
d: f__Prevotellaceae
e: f__Rikenellaceae
f: f__Tannerellaceae
g: f__Gastranaerophilales
h: o__Gastranaerophilales
i: c__Vampirivibrionia
j: f__Deferribacteraceae
k: o__Deferribacterales
l: c__Deferribacteres
m: f__Desulfovibrionaceae
n: o__Desulfovibrionales
o: c__Desulfovibrionia
p: f__Enterococcaceae
q: f__Lachnospiraceae
r: o__Lachnospirales
s: f__Ruminococcaceae
t: f__UCG_010
u: o__Oscillospirales
v: f__Anaerovoracaceae
w: f__Peptostreptococcaceae
x: o__Peptostreptococcales_Tissierellales
y: c__Clostridia
z: o__Veillonellales_Selenomonadales
a0: c__Negativicutes
